# Bioprosthetic valve thrombosis after transcatheter aortic valve replacement and pulmonary embolism due to heparin-induced thrombocytopenia: a case report

**DOI:** 10.3389/fcvm.2023.1164432

**Published:** 2023-08-08

**Authors:** Loïc Faucher, Benjamin Marchandot, Adrien Carmona, Mickael Ohana, Antonin Trimaille, Olivier Morel

**Affiliations:** Hôpital Civil, Strasbourg, France

**Keywords:** TAVR—transcatheter aortic valve replacement, heparin-induced thrombocytopenia, thrombosis—etiology, bioprosthetic valve dysfunction, argatroban, pulmonary embolism, intensive unit care

## Abstract

**Background:**

Bioprosthetic valve thrombosis is a complication of transcatheter aortic valve replacement (TAVR). It is believed to be platelet independent, mainly driven by contact phase activation, and more likely to be targeted by oral anticoagulant (OAC).

**Case summary:**

We report case of an 86-year-old man with history of TAVR, who presented an early TAVR aortic valve thrombosis occurring in the context of heparin-induced thrombocytopenia (HIT) and pulmonary embolism. The patient rapidly recovered and was discharged 17 days after readmission. OAC by Coumadin was administered for 3 months. Chest tomography after 3 months showed the disappearance of the hypoattenuated leaflet thickening.

**Discussion:**

Although HIT has been fully described and is known for being a prothrombotic disorder, this is the first case report of aortic valve thrombosis after TAVR due to HIT. HIT is rare but possibly lethal. Diagnosis is based on pre-test probability evaluation with the 4T clinical score and confirmation with laboratory evidence of anti-PF4/heparin complexes and positivity of a functional test. Management of HIT is based on heparin discontinuation, and treatment of thrombotic complication with direct anti-IIa inhibitor or anti-Xa inhibitor. According to our knowledge, this case represents the first report of bioprosthetic valve thrombosis after TAVR due to HIT.

## Introduction

Bioprosthetic valve thrombosis occurs in a sizeable proportion (4%–15%) of patients with transcatheter aortic valve replacement (TAVR) (1, 2). It is believed to be platelet independent, mainly driven by contact phase activation, and more likely to be targeted by oral anticoagulant (OAC). Here, we report the case of a bioprosthetic valve thrombosis due to a heparin-induced thrombocytopenia (HIT).

This is the first report of an early TAVR aortic valve thrombosis occurring in the context of HIT.

## Case presentation

An 86-year-old patient was referred to the emergency department with dyspnea, dry cough, and chest pain (Table 1). The physical examination was unremarkable, with an initial blood pressure (BP) of 130/66 mmHg, pulse of 95 beats/min, oxygen saturation of 92%, and no sign of heart failure. Electrocardiogram at admission was normal. Dimer-d were elevated (>20,000 µg/L) and a computed tomography (CT) pulmonary angiogram evidenced proximal bilateral acute pulmonary embolisms without signs of acute right ventricular failure. Laboratory data revealed thrombocytopenia with a reduced platelet count of 55 × 10^9^/L. Unfractionated heparin (UFH) was administrated together with oxygen therapy (2 L/min). Biological assessment performed 8 h after UFH onset showed a large drop in platelet count of 38 × 10^9^/L.

**Table 1:** Timeline.

**14/12/2020**	Non-ST elevation myocardial infraction treated by left anterior descending and first diagonal stenting
**23/09/2021**	Transcatheter Aortic Valve Replacement with a Sapien 3 23mm
**05/10/2021**	Emergency department with dyspnea, a dry cough and chest pain. Laboratory data revealed thrombocyteopenia. Computed tomography (CT) scan confirmed diagonsis of acute valve thrombosis. Treatment with Argatroban was started.
**06/10/2021**	Laboratory assay confirmed Antibodies anti-PF4 positive
**11/10/2021**	Coumadin initiation after platelet count was superior to 150 *109/L.
**21/10/2021**	Discharge on Vitamin K agonist

**Figure 1:**
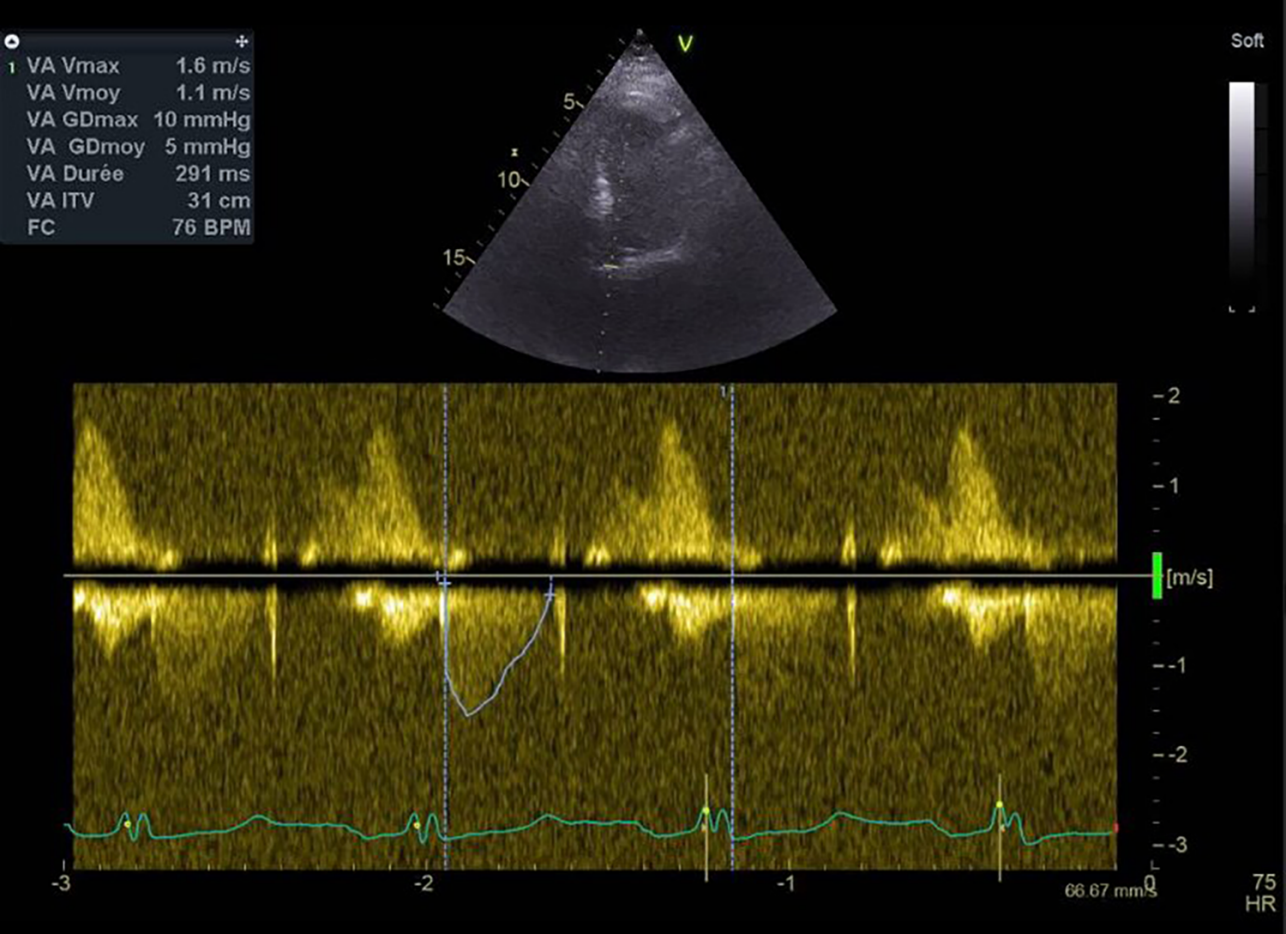
Mean transprosthetic gradient immediately after TAVR, measured by TTE.

Two weeks before admission to the emergency department, the patient underwent TAVR for symptomatic severe aortic stenosis. The TAVR procedure was performed with a Sapien 3 23 mm under anticoagulant therapy (12,500 UI of UFH, with an activated clotting time of 225). Heparin was antagonized by protamine at the end of the procedure. The mean transprosthetic gradient assessed by transthoracic echocardiography (TTE) was 5 mmHg (Figure 1), and the patient was discharged home on day 4 under dual antiplatelet therapy with aspirin and clopidogrel in the context of recent percutaneous coronary intervention (PCI). Platelet count at discharge was 120 × 10^9^/L.

The patient had a previous history of non-ST-elevation myocardial infarction treated by left anterior descending and first diagonal stenting (done on 2020), hypertension, and diabetes mellitus. Treatment of the patient at admission included dual anti-aggregation with aspirin and clopidogrel, ramipril, and antidiabetic treatment with metformin and insulin. Pre-TAVR cardiac ultrasound found a left ventricular ejection fraction at 63% without disorder of parietal kinetics. The mean transaortic gradient was 42 mmHg. The aortic valve area was estimated at 0.83 cm^2^ (indexed aortic valve area 0.43 cm^2^/m^2^).

**Figure 2:**
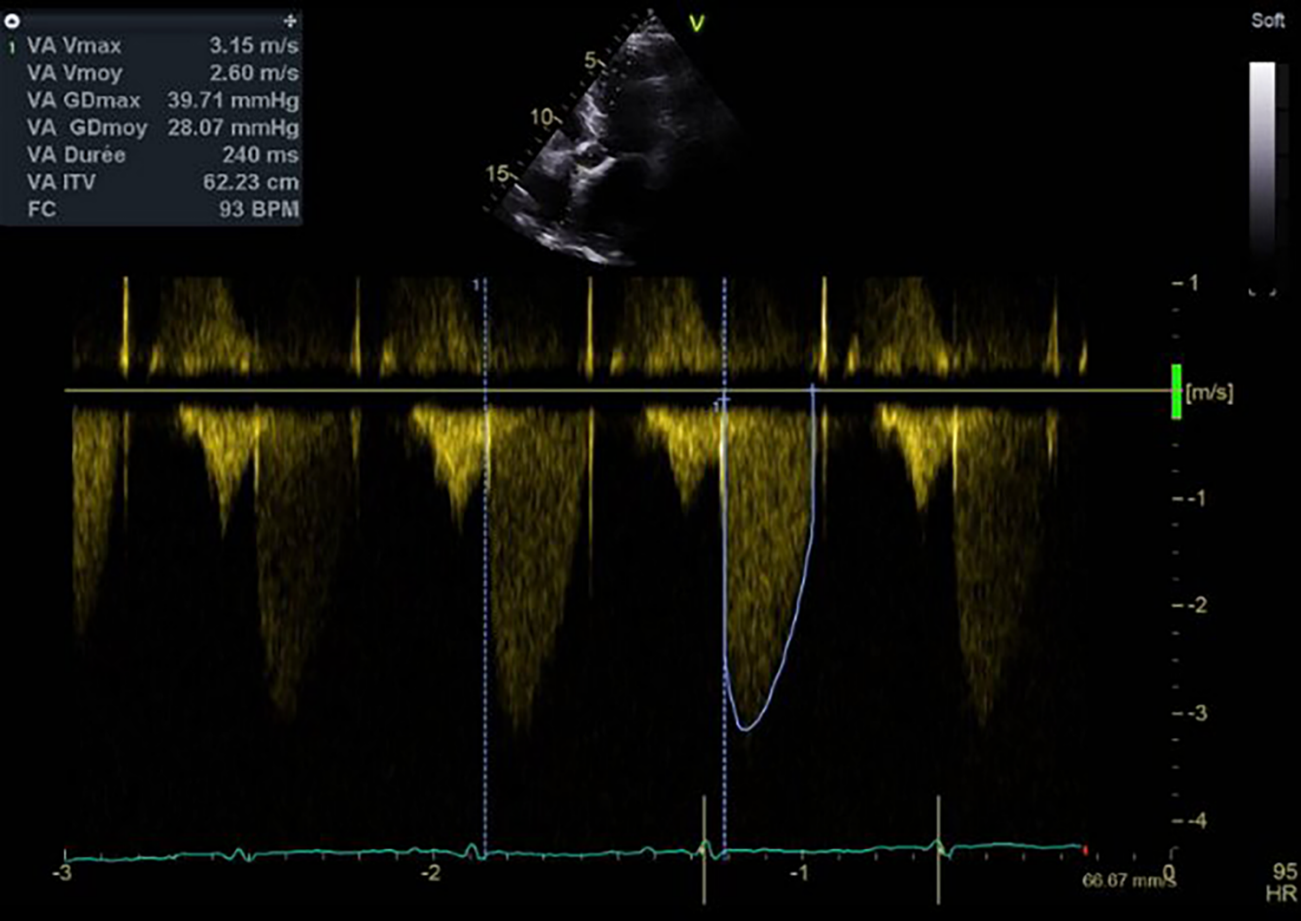
Mean transprosthetic gradient at diagnosis of HIT, assessed by TTE.

**Figure 3:**
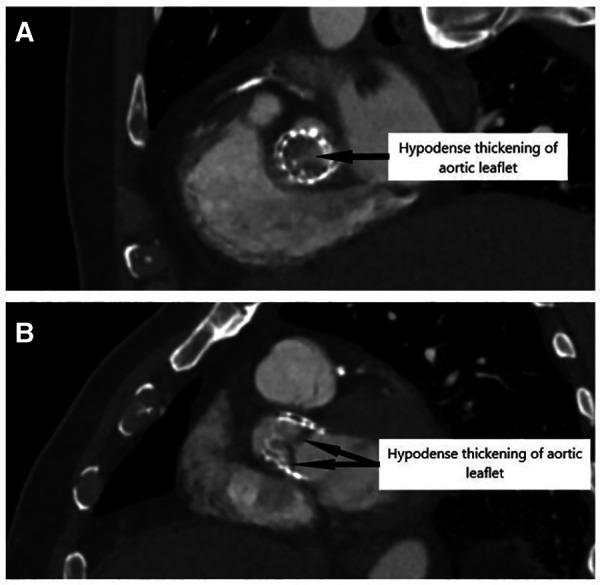
Cardiac tomography that shows a hypodense thickening of aortic cusps, confirming diagnosis of valve thrombosis, in sagittal (**A**) and transversal (**B**) views.

A diagnosis of HIT was suspected. The 4T score was calculated at seven points (Table 2). UFH was immediately stopped and switched for argatroban. The anti-PF4 antibody assay came back positive (optical density at 2.4 for a positivity threshold at 0.17). A functional test was performed to confirm the diagnosis, with a platelet activation test coming back positive. By TTE, aortic valve thrombosis was suspected with significant elevation of the mean transprosthetic gradient at 28 mmHg and reduced leaflet motion of the right coronary cusp (Figure 2). No evidence of right ventricular dilatation or acute heart failure was found. Cardiac tomography was performed that confirmed the diagnosis of bioprosthetic valve thrombosis with a hypodense thickening of the aortic cusps (Figure 3). A venous Doppler evaluation revealed a recent left sural vein thrombosis. Treatment with argatroban was continued. Clopidogrel was discontinued with continued aspirin therapy.

**Table 2:** Pretest scoring system for HIT: the 4 T'score.

4T’s	2 points	1 point	0 point
Thrombocytopenia	Platelet count fall >50% and platelet nadir ≥20	Platelet count fall 30–50% or platelet nadir 10–19	Platelet count fall <30% or platelet nadir <10
Timing of platelet count fall	Clear onset between days 5–10 or platelet fall ≤1 day (prior heparin exposure within 30 days)	Consistent with days 5–10 fall, but not clear (e.g. missing platelet counts); onset after day 10‡; or fall ≤1 day (prior heparin exposure 30–100 days ago)	Platelet count fall <4 days without recent exposure
Thrombosis or other sequelae	New thrombosis (confirmed); skin necrosis; acute systemic reaction postintravenous unfractionated heparin (UFH) bolus	Progressive or recurrent thrombosis; non-necrotizing (erythematous) skin lesions; suspected thrombosis (not proven)	None
Other causes for thrombocytopenia	None apparent	Possible	Definite

**Figure 4:**
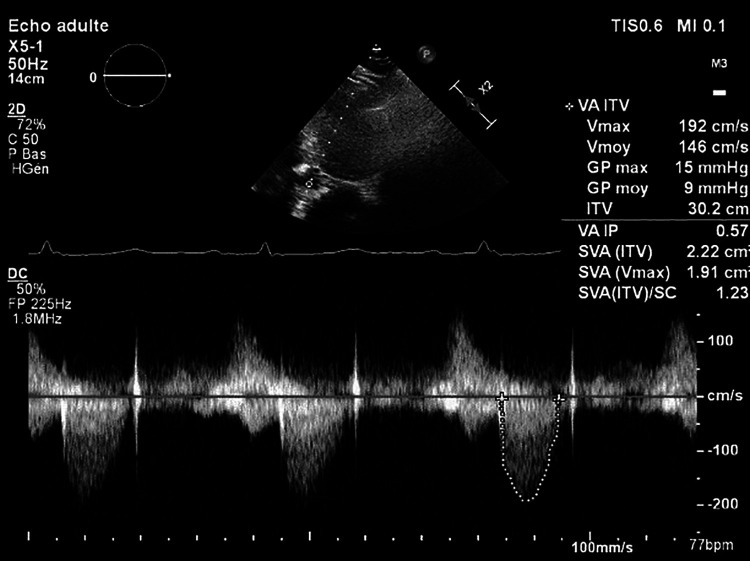
Mean transprosthetic gradient at discharge, after treatment with argatroban and initiation of Coumadin.

Cardiac echocardiography after 1 week of treatment with argatroban revealed a significant decrease in the mean transprosthetic gradient of 9 mmHg (Figure 4). OAC by Coumadin was started at day 7 once the platelet count was superior to 150 × 10^9^/L.

The patient rapidly recovered and was discharged 17 days after readmission. OAC by Coumadin was administered for 3 months. Chest tomography after 3 months showed the disappearance of the hypoattenuated leaflet thickening (HALT). Clinically, the patient is asymptomatic, NYHA 1. The latest biological results showed the platelet count to be 173 × 10^9^/L (November 2022). Regular ultrasound follow-up is performed. The parameters remain stable with an average gradient at 9 mmHg more than a year after discharge.

## Discussion

There are two types of HIT. Type 1 occurs within the first 2 days after exposure to heparin, and the platelet count normalizes even with continued heparin therapy. Type 1 HIT is a nonimmune disorder that results from the direct effect of heparin on platelet activation (2, 3).

Type 2 HIT is an immune-mediated disorder that typically occurs 4–10 days after exposure to heparin. Pathophysiological mechanisms involved the formation of an immunocomplex consisting of an autoantibody against the Platelet Factor 4 antibodies (PFA)–heparin complex. PF4 antibodies are thought to activate platelets through FcgRIIa receptors, leading to drastic platelet activation, externalization of procoagulant aminophospholipids such as phosphatidylserine, and the release of microparticles (MPs) enriched in phosphatidylserine and tissue factor. The interaction between PFA antibodies and FcgRIIa receptors was also demonstrated to be an important mechanism involved in monocytic and endothelial cell activation. Activated platelets, endothelium, and leukocytes together with derived MPs provide an additional surface enabling the formation of the characteristic enzyme complexes tenase and prothrombinase leading to thrombin generation and coagulation.

Female sex, age <40 years, and the use of UFH (in comparison with low molecular–weight heparin) are well known risk factors of HIT (3, 4).

In clinical practice, HIT diagnosis is based on clinical and biological features. The 4T clinical scoring system is the most used; the score is a cumulative measure that incorporates quantification of thrombocytopenia, thrombosis, and timing of heparin therapy as well as searches for other causes of thrombocytopenia. Each item is scored 0–2 points. The score is the sum of the values of each category. HIT probability is low if the 4T score is between 0 and 3 points, moderate between 4 and 6 points, and elevated if it is higher than 6 points. Laboratory evidence of anti-PF4/heparin complexes is essential for HIT diagnosis and relies on the detection of HIT antibodies by immunoassays combined with platelet activation assays.

Treatment of HIT should start at the time of diagnosis suspicion and relies on heparin eviction when pre-test probability of HIT is moderate or high (i.e., 4T score ≥4). Argatroban is a direct thrombin inhibitor that is approved for treating thrombosis-complicating HIT. Danaparoid is a mixture of non-heparin anticoagulant glycosaminoglycan that inhibits long-acting antithrombin-dependent anti-Xa activity, and is approved for this indication (4).

Bioprosthetic valve thrombosis occurs in a sizeable proportion of TAVR patients (4%–15%). Valve thrombosis should be suspected when the mean transvalvular gradient is ≥20 mmHg or increased more than 10 mmHg when compared with baseline. Diagnosis is confirmed by multidetector computed tomography via the detection of HALT and a concomitant reduction in leaflet motion (RELM) (5). In general, thrombus formation is believed to result from the complex interplay between calcifications, the native valve potentially being enriched in prothrombotic factors, flow stasis, procedural factors, and valve type (5).

In the context of TAVR, we and others (6) have demonstrated that the development of bioprosthetic valve thrombosis is mainly platelet independent, consistent with a primary contact phase activation and more likely to be targeted by OAC. Bioprosthetic valve thrombosis has been fully described after TAVR (5). Scott et al. reported the case of a 74-year-old man with the antecedent of HIT who underwent TAVR, using bivalirudin in substitution of UFH (7).

HIT is now a well-recognized diagnosis. If bioprosthetic valve thrombosis has already been described after cardiac surgery, this is the first case described after TAVR. With the increase in the number of TAVR procedures performed worldwide, this case is a reminder of the importance of investigating the diagnosis of HIT in cases of early valve thrombosis after TAVR. Similarly, any thromboembolic episode occurring after TAVR should be investigated for HIT.

## Conclusion

Thrombosis manifestations occurring in heparin-induced thrombocytopenia have been fully described. Our case highlights the possibility of heparin-induced thrombocytopenia as a cause of valve thrombosis after percutaneous valve implantation.

## Data Availability

The original contributions presented in the study are included in the article/Supplementary Material, further inquiries can be directed to the corresponding authors.
